# Imaging patients with stable chest pain special feature: introductory editorial

**DOI:** 10.1259/bjr.20209005

**Published:** 2020-08-19

**Authors:** Matthijs Oudkerk, Edwin JR van Beek

**Affiliations:** 1 University of Groningen, Groningen, Netherlands; 2 Institute for Diagnostic Accuracy, Groningen, Netherlands; 3 Edinburgh Imaging, University of Edinburgh, Edinburgh, UK

## Chest pain in medical research perspective: 700 years of trial and error

The coronary circulation was discovered in the 13th century by Ibn al Nafis (1213–1288).^1^ In Damascus, Syria, he published the *Kitab Mujiz – The concise book (1250*) with the following description: *“the nourishment of the heart is from the blood that goes through the vessels that permeate the body of the heart.”* This was a precise functional discovery of the coronary blood circulation specially conditional in relation to chest pain. Leonardo da Vinci (1452–1519) presented the first morphological drawing of the coronaries of animals in his famous anatomical sketches (c 1485 Florence).^2^ In Padua, Vesalius (1514–1564) published his classical work *De Humani Corporis Fabrica* (1543) with the first anatomical drawing of the coronaries from autopsy studies of the human body.^3^ Also, at the illustrious University of Padua, William Harvey discovered the functional system of blood circulation in 1628 laying the foundations of modern experimental medicine.^4^ On 21 July, 1768 William Heberden delivered a lecture before the Royal College of Physicians of London on a disorder of the breast,^5^ a form of chest pain which he describes as follows: “*But there is a disorder of the breast marked with strong and peculiar symptoms, considerable for the kind of danger belonging to it, and not extremely rare, which deserves to be mentioned more at length. The seat of it and the sense of strangling and anxiety with which it is attended, may make it not improperly be called angina pectoris"*. After his masterly description of the chest disorder which Heberden called angina pectoris, it took more than 150 years before the syndrome was linked to its true etiology.

In the meantime Morgagni (Padua, 1761) published on calcifications in the coronary arteries of an old male.^6^ For a long period, this led to the hypothesis of Jenner that calcifications were the origin of angina pectoris as has been published in 1793 (London).^7^ But as a result of the animal experiments of John Erichsen in 1842 (London),^8^ in which he ligated the proximal coronary arteries it became clear that occlusion of the coronary arteries may cause a fatal cardiac event: “…*that the arrest of the coronary circulation produces a speedy cessation of the heart’s action. Secondly, that an increase in the quantity of blood sent into, or retained in the muscular fibre of the heart, produces a corresponding increase in the activity of that organ.”*

This was the beginning of coronary ligation experiments for physiological studies of myocardial perfusion to the present day. In 1907, the visualisation of the coronary arteries by X-ray was published in an atlas composed from the analysis of human cadavers by Jamin and Merkel.^9^

After this long period of hypothesising and speculating by the medical scientific community on the origin of angina pectoris, in 1928 Chester Keefer and William Resnik stated that from all available evidence angina pectoris is caused by anoxemia of the myocardium.^10^ This publication triggered the interest to visualise the coronary arteries in the living human body. Rosthoi performed the first left ventriculography and coronary visualisation in an animal experiment in 1933.^11^ Radner from Sweden realized the first “*in vivo*” coronary angiogram by direct sternal puncture of the ascending aorta in the year 1945.^12^ After this very drastic approach followed a period when catheters were inserted in the arterial system from the femoral artery and contrast was injected in the aorta at the level of the aortic root, while selective coronary contrast injection in the coronaries was regarded as a potential cause of iatrogenic mortality and was avoided. On 30 October, 1958, during such an angiographic procedure performed by Sones, the catheter accidentally stuck in the main coronary stem and the first selective cine frame coronary arteriogram was recorded.^13^ To the astonishment of the medical community, the patient survived without any sequalae. This positive outcome promoted diagnostic coronary angiography for the diagnosis of morphological coronary changes as a proof for the origin of chest pain, because those changes were regarded to be the key etiology for hypoxemia of the myocardium. Diagnostic coronary angiography and percutaneous transluminal angiography were developed through the 1960s and 1970s by the radiological research of Dotter^14^ with diminishing procedure related complication and mortality rates. As radiologist Gruentzig performed the first percutaneous transluminal coronary angioplasty on 16 September, 1977 in Zurich Switzerland.^15^ The nonsurgical treatment of coronary stenosis by angioplasty unleashed the medical specialty of the interventional cardiologist focusing on the treatment of coronary stenosis by angioplasty and stenting as the solution to prevent myocardial ischemia and hypoxemia. However, this monocausal approach led to substantial overdiagnosis and overtreatment of coronary artery stenosis.

In 1988, the first continuously rotating CT systems became clinically available with rotation times lower than 1s.^16^ In 2001, 4-Multi Detector CT Systems became available with rotation times lower than 500 ms enabling the noninvasive visualisation of the coronaries.^17^ It took almost 20 years of further development of coronary CT angiography before it could establish a major position in the diagnostic work-up of patients with chest pain. Systematic population research with CT examinations showed that the majority of people will develop coronary artery calcification and stenosis with ageing without any consequences for the oxygenation of the myocardium. This led to the insight that the majority of coronary stenoses do not need treatment, because there is no effect on the perfusion of the myocardium by flow redistribution and collateral flow mechanisms. This in turn resulted in a sharp rise of noninvasive diagnostic CT evaluation of the coronaries in patients with chest pain to rule out the many different pathologies that were already described by Heberden in 1768. Moreover, noninvasive coronary CT angiography is now used to diagnose and select the coronary pathologies that need (immediate) revascularization therapy by percutaneous coronary intervention or coronary artery bypass grafting. We reached the point that invasive diagnostic coronary angiography is hardly ever indicated and invasive procedures should be reserved for coronary treatment procedures alone.

With the recent publication of the SCOT-HEART study,^18^ a new era of noninvasive coronary imaging opens up. The study proved that replacing the diagnostic work-up by noninvasive coronary CT imaging in patients with chest pain, as a first-line modality, results in a substantially lower mortality rate compared to a diagnostic strategy without this noninvasive diagnostic modality. Only 6 months after the SCOT-HEART publication, the final evidence was published that noninvasive myocardial perfusion MR can rule out all patients with significant coronary pathology but without any impact on myocardial bloodflow.^19^

This *BJR* special feature is dedicated to these landmarks and aims to open up the horizons of the many new applications that will dramatically change the current medical practice. The special feature includes an overview of the SCOT-HEART trial and its impact on CCTA for patients with stable ischemic heart disease^20^, as well as a review of cost-effectiveness for imaging stable ischemic disease^21^. Furthermore, the collection gives a detailed review of the potential for functional coronary and cardiac CT imaging beyond the evaluation of the coronary artery lumen^22^, the potential role of noncardiac findings in risk stratification^23^ and the role of machine learning to drive forward enhanced imaging data analysis.^24,25^ In addition, the role of MRI for the assessment of chest pain is of great importance^26–28^, as is the role of imaging in the evaluation of heart valve disease^29^. Lastly, novel methods for the evaluation of heart viability and coronary artery disease^30^, with a focus on developing ways to assess vulnerable plaque^31,32^, are coming to the fore and are reviewed.

This *BJR* special feature marks the moment of publication of the first hard evidence that noninvasive coronary CT imaging in patients with chest pain saves lives compared to current medical practice and at the same time is a lot less harmful for the patient, costs less and is more effective. The special feature appears also at the moment of 125 years of publishing radiological research in *BJR. BJR* is the oldest radiology journal in the world and this excellent collection of articles demonstrates that the journal remains at the cutting edge of radiological research publishing. This fascinating collection will be of value to any medical professionals interested in stable chest pain. The Guest Editors would like to thank all of the authors for contributing their work as well as the expert reviewers who helped review it. We hope our readers enjoy the collection!
